# Prevalence and factors associated with dietary supplement use among Bangladeshi public university students: A cross-sectional study

**DOI:** 10.1371/journal.pone.0276343

**Published:** 2022-10-17

**Authors:** Md. Abu Tareq, Umme Habiba Emi, Md. Hasan Al Banna, Humayra Rezyona, Abdul-Aziz Seidu, Mohammad Tazrian Abid, Justice Kanor Tetteh, Mst. Sadia Sultana, Satyajit Kundu, Md. Hasanuzzaman, Shuvajit Mondal, Moumita Mandal, Md. Shafiqul Islam Khan

**Affiliations:** 1 Department of Food Microbiology, Faculty of Nutrition and Food Science, Patuakhali Science and Technology University, Patuakhali, Bangladesh; 2 Faculty of Nutrition and Food Science, Patuakhali Science and Technology University, Patuakhali, Bangladesh; 3 Department of Food and Nutrition, College of Home Economics, Azimpur, Dhaka, Bangladesh; 4 Centre for Gender and Advocacy, Takoradi Technical University, Takoradi, Ghana; 5 College of Public Health, Medical and Veterinary Sciences, James Cook University, Townsville, Australia; 6 Department of Population and Health, University of Cape Coast, University Post Office, Cape Coast, Ghana; 7 Department of Public Health and Informatics, Jahangirnagar University, Savar, Dhaka, Bangladesh; 8 School of Public Health, Southeast University, Nanjing, China; 9 Department of Environmental Sanitation, Faculty of Nutrition and Food Science, Patuakhali Science and Technology University, Patuakhali, Bangladesh; Noakhali Science and Technology University, BANGLADESH

## Abstract

**Introduction:**

The usage of dietary supplement (DS) such as vitamins, minerals, and fish oil has expanded, but there is limited data on their use by sub-populations such as university students. The study was aimed to investigate the prevalence of DS use among Bangladeshi university students and its associated factors.

**Methods:**

A cross-sectional survey of 390 students was conducted from two public universities from Barishal Division in Bangladesh using a structured questionnaire with 72 questions divided into five sections: sociodemographic, knowledge, opinions, and attitudes, types of DS, reasons and sources for using DS, and adverse reactions after taking DS. Descriptive statistics and logistic regression were utilized to estimate the results.

**Results:**

Among all the students, 15.6% students were using DS where only 7.7% of them used DS according to physicians’ recommendation. Additionally, students used DS for general health and well-being, weight gaining and as a source of energy for physical and sporting activities, etc. The use of DS was significantly associated with female sex (AOR = 5.44, 95% CI: 2.18–13.52), ≥25 years age (AOR = 0.08, 95% CI: 0.01–0.67), underweight (AOR = 5.86, 95% CI: 1.95–17.62), having major illness (AOR = 6.99, 95% CI: 1.98–24.70) and good knowledge of DS (AOR = 2.64, 95% CI: 1.23–5.64).

**Conclusion:**

This study provides new findings on DS use and its correlates in Bangladeshi students which may be used by the policymakers to improve DS usage among students. Adaptation of an appropriate program is recommended to educate students on proper and safer ways of using DS.

## Introduction

In the late 70’s and 80’s, diet intended to add more nutritional value to normal diet became very popular and widespread across the world [1, 2]. This follows public claims by Nobel-Laureate Linus Pauling that more doses of ascorbic acid above the recommended dietary allowance could treat or prevent common cold, flu and cancer [3]. The widespread usage especially in the USA led to the USA Congress pass a law in 1994 called Dietary Supplement Health and Education Act (DSHEA) to define and regulate its usage with special focus on safety, quality and efficacy [1]. Dietary supplement (DS) has been defined to include all food or dietary products that are intended to add more dietary value to supplement normal diets [4]. The DSHEA (1994) of the USA defined dietary supplement to include all food products that contains high volumes of dietary ingredients such as vitamins, minerals, herbs, amino acid, concentrates, metabolites, melatonin, bone meal, fish oil and etc. [1]. Tasfia et al. (2020) [5] has further explained that DS “is generally a calculated planning to parallel the diet which contains mostly vitamins and minerals and a lesser extent amino acid, herbs and fish oils which serves as an important source of essential nutrients and often provides solution to daily nutritional deficits and helps to fight or prevent diseases and improve the overall health conditions of individuals”.

The DSHEA of the USA has been adapted by many countries all over the world due to the widespread usage of DS [6, 7] and Bangladesh is no exception. The Food Safety Act 2013 of the People’s Republic of Bangladesh is mandated to regulate the use of DS in Bangladesh and their work is especially very crucial today due to reports of high DS usage in Bangladesh as a result of reported increasing high prevalence of micronutrients deficiencies in the general population [5, 8]. The micronutrients depletion situation in Bangladesh is growing day by day and this could be a source of motivation or reason for high DS patronage. Unlike in the USA and many developed countries in Europe where extensive research has been conducted to examine factors associated with DS usage to inform policy and regulations, very little has been done in Bangladesh [5, 7, 9].

Factors associated with dietary supplement use as reported in studies conducted in developed countries [1, 4, 10] which include; adults or older men and women, persons who are physically active, persons less likely to smoke, persons with low body mass index (BMI), pregnant women, persons with high educational status and persons with high socioeconomic status in comparison with non-users. However, it is unclear how these factors apply in Bangladesh due to discrepancies in different factors including socio-cultural and socioeconomic and socio-demographic which are present in these study settings. Few studies conducted in Bangladesh previously have concentrated on only females [5, 9] with the reason being that females are more prone to suffer from nutritional deficiencies than men due to their female reproductive system, low social status, poverty and lack of education [8]. However, studies in other parts of the world have reported high DS patronage among males mostly for sports and physical activity purposes and also for the boosting of the body’s immune system [4, 10, 11]. It will therefore be informative to examine the prevalence of DS usage among both male and females with the same socio-demographic characteristics, especially in Bangladesh.

For better regulation of DS usage in Bangladesh, it is paramount to know how prevalent the usage of DS is and the factors associated with its usage. Previous studies have also highlighted the need for further investigation into DS use in Bangladesh [7]. This study therefore sought to employ a cross-sectional study design to investigate the prevalence of dietary supplement use and its associated risk factors among Bangladeshi university students to assist policy makers towards its proper regulation in Bangladesh.

## Methods and materials

### Study settings

This research was conducted at two Bangladeshi public universities, which are situated in Barishal division, a coastal region in the southern part of Bangladesh. Barishal is approximately 238 km away from Dhaka, the capital city of Bangladesh. There are two public universities in this division, namely, Barishal University (BU) and Patuakhali Science and Technology University (PSTU). BU, the only public university in the suburbs of Barishal district and located about 2 kilometers from the city’s center near Karnakathi on the eastern bank of the Kirtonkhola river. PSTU is the first public university of Barishal division, located at dumki upazila, Patuakhali district. This area is about 38 kilometers south of Barishal city and 270 kilometers from the capital city. PSTU (in main campus) has six residence halls (dormitories) which can accommodate 3000 students (two are for females and four are for males). BU has three dormitories (two for females and one for males) with nearly 2500 students. In Bangladesh, studies regarding dietary supplements and their contributors have been conducted at several universities in the south-east and a few districts in the southern. Nevertheless, no research has been conducted on the southern coastal universities that could be replicated on this socio demographic perspective of this region. Therefore, this area was considered suitable to investigate the intensity of dietary supplement use among university students where a diverse culture, sociodemographic, and other characteristics were greatly affecting the use of these supplements.

### Study design, participants and sampling

A cross-sectional study was carried out among Bangladeshi university students to explore the prevalence of dietary supplement use and its associated risk factors. The study period was from October 2021 to March 2022. The inclusion criteria for the study participants were: (i) students aged 18 years or above and (ii) Bangladeshi by birth. We calculated a minimum sample of 317 participants using the formula of single sample proportion test, n = z^2^pq/d^2^. Where, n = desired sample size, z = confidence level (1.96), p = estimated proportion of an attribute that is present in the population (0.41) according to the prevalence of DS use in Bangladesh [7], q = (1–0.41) and d = margin of error (0.05). By considering a 10% non-response rate, an optimal sample size of 348 was determined. To obtain more precious findings and to avoid any missing data sets, we finally included a sample of 390 students in this study. We chose three faculties out of six from the BU using a simple random lottery method. Similarly, four faculties were selected from the eight faculties at PSTU. Participants were recruited randomly from the selected faculties.

### Survey tool and measurement

The questionnaire included 72 questions, divided into 5 major sections. The first section contained information on respondents’ sociodemographic (such as age, gender, residency, education, faculty, job nature and monthly income) and lifestyle characteristics such as self-rated BMI and health status, smoking habits, and level of physical activity.

The second section included questions concerning knowledge about dietary supplements which was retrieved from the previous study [12, 13]. However, the knowledge about DS was assessed using the 5-point Likert 12 questions in the context of safety of dietary supplements, as it is food items, natural ingredients, about the efficacy, disease prevention and effectiveness. The options ranged from “strongly agree” to “strongly disagree” (strongly agree = 0 to strongly disagree = 4). "Strongly disagree" was intended as the most preferable response in all questions. It’s usual for supplement providers to claim the safety of their products by using phrases such as "dietary supplements are just foods" or “dietary supplements made from natural ingredients or herbs are safe", regardless of whether or not their products are properly regulated. Clearly, "strongly disagree" is the preferred perception in this situation. Similarly, it is not necessary to avoid all food additives, since the amounts are strictly regulated to be below the acceptable daily intakes; therefore, we can’t not avoid them completely. Food additives, regardless of their kind and amount, are believed to cause cancer by the majority of consumers. Moreover, considering our current situation in Bangladesh, we set "strongly disagree" as the most preferable answer for the remaining topics [12].

In part three, attitudes about DS were assessed using the 5-point Likert 6 questions which was derived from a study conducted in Bangladesh [9] in the context of favorable effects of DSs and the prevention of cancer and chronic illness when used regularly. The options of the Likert questions ranged from "strongly agree" to "strongly disagree” and score for each option was assigned 0 to 4 for strongly disagree to strongly agree. “Strongly agree" was intended to be the preferred choice for each question. All the questions involving in the attitude section indicated positive responses as true; that’s why strongly agree is regarded as the acceptable answer in this study. In the fourth section, respondents were asked about the estimated intake of DS types, the reasons for using DS, and the sources of DS. Finally, the respondents were asked about adverse reactions after taking DS.

### Reliability and validity of the questionnaire

In addition to being developed in English, the questionnaire was translated into local language (Bengali) by a few experts in both languages before initiating the data collection process. A piloting and statistical validation process were conducted on the questionnaire. Besides, interviewers were rigorously trained in accordance with detailed guidelines before the final investigation was conducted. The questionnaire was pre-tested among 30 students (15 students per university) to determine its clarity, practicality and relevance to the participants as well as to identify ambiguities in the items that could compromise its validity and reliability. Further necessary modifications were incorporated in the questionnaire according to the result gathered from the piloting. The pre-tested results were not included in the final analysis. The internal consistency for knowledge (Cronbach’s α = 0.74) and attitude (Cronbach’s α = 0.71) parts were within acceptable limit [14].

### Operational definition of DS and dietary supplement users

Dietary supplements are governed by law in the USA and European countries; they are very well defined, but not in Bangladesh. Throughout this study, dietary supplements were defined as any pill, capsule, coated tablet, drop, powder, or beverage that was marketed primarily for its nutritional value or putative health-promoting function and had one or more ingredients (such as vitamins, amino acids, etc.) intended to supplement a person’s diet and were not classed as food [15]. Dietary supplement users are defined as those who have recently taken a supplement in the last three months.

### Data collection procedure

Data for this study were collected using a hard copy pretested structured questionnaire through the conduct of face-to-face interviews. Since some of those data were sensitive, we recruited women research assistants (RAs) for female participants with required experience. An initial two-day training session was taken place prior to the interview to ensure that the RAs were familiar with the questionnaire. Then, appropriate respondents who provided their informed consent to participate were approached by RAs. All the study procedures adhered to the Declaration of Helsinki and its later amendment. It took about 25 to 30 minutes to complete each interview.

### Data analyses

Descriptive statistics with the indication of frequency and percentage was employed to determine the demographic characteristics of the participants, the types and frequency of diet supplements used, knowledge, attitude toward diet supplements usage and reasons for using as well as the types and sources of it. Participants level of knowledge and attitudes regarding DS were calculated according to the mean value score. Individual who obtained a score of above mean for knowledge and attitudes test was considered good knowledge and positive attitudes regarding DS, respectively. To identify factors associated with the use of DSs, a binary logistic regression model was used. The variables related to socio-demographics, socioeconomics, and other factors, and the level of knowledge (good and bad) and attitudes (positive and negative) regarding DS were all included in both univariate (unadjusted) and multivariate (adjusted) logistic regression model. The variance inflation factor (VIF) and tolerance were used to test for multicollinearity among the independent variables. The adjusted model had a mean VIF of 1.463 (min VIF = 1.056, max VIF = 2.432), though it is generally considered acceptable for a mean VIF of less than ten [16, 17]. Odds ratios (95% confidence interval, CI) were calculated to assess the association between independent variables and dependent variables (use of DS). Statistical significance was determined by a p value less than 0.05. All statistical analysis was performed by statistical package for the social science (SPSS) software (IBM version 23.0, Armonk, NY, USA).

### Ethics statement

The study protocol was evaluated and approved by the Research Ethical Committee (REC) of the Department of Food Microbiology, Patuakhali Science and Technology University, Bangladesh [approval number: FMB: 13/09/2021:04]. Written consent was taken from each of the surveyed individuals after discussing the purpose of the study and the confidentiality of their personal information.

## Results

### Socio-demographic, behavioral and diet-related characteristics of study participants

Respondents’ socio-demographic, behavioral and diet-related characteristics are highlighted in Table 1. Of 390 respondents, more than half (55.1%) were female and rest of them were male (44.9%). Nearly three-quarters of the respondents (71.0%) were between 21 to 24 years old, with a mean age of 22.17 (SD = ± 1.81) years. The majority of the respondents (91.8%) were studied in undergraduate level, in which one-third (35.4%) were in their first year of under graduation. Over two-thirds of the respondents (69%) reported normal body mass index (BMI). Half of the respondents (51.3%) reported their self-related health status as good. Moreover, more than three-quarters of the respondents (80.8%) hadn’t followed a specific diet, but agreed (76.7%) that dietary supplements were beneficial to health (Table 1).

**Table 1:** Socio-demographic, behavioral and diet-related characteristics of study participants (N = 390).

Characteristics	Frequency	Percentage
**Age (years)**		
18–20	71	18.2
21–24	277	71.0
25 and above	42	10.8
Mean ± SD 22.17 ± 1.81		
**Gender**		
Male	175	44.9
Female	215	55.1
**Household income (Bangladeshi taka/month)**		
0–15000	58	14.9
15001–30000	171	43.8
30001–45000	90	23.1
45001-above	71	18.2
**Study year**		
1^st^ year	138	35.4
2^nd^ year	84	21.5
3^rd^ year	72	18.5
4^th^ year	64	16.4
Above honors	32	8.2
**Current marital status**		
Married	30	7.7
Unmarried	360	92.3
**Suffer from a major Illness**		
Do not suffer	369	94.6
Suffer	21	5.4
**Self-Related BMI**		
Normal	269	69.0
Overweight or obese	85	21.8
Underweight	36	9.2
**Self-rated health status**		
Excellent	55	14.1
Good	200	51.3
Fair	78	20.0
Below	57	14.6
**Type of diet followed**		
High Protein Diet	34	8.7
Low fat diet	41	10.5
No specific diet followed	315	80.8
**Smoking status**		
Current Smoker	29	7.4
Ex-smoker	15	3.8
Never smoking	346	88.7
**Physical Activity**		
No activity	192	49.2
<30 minutes/day	129	33.1
>30 minutes per day	69	17.7
**Diagnosis of chronic disease**		
Yes	40	10.3
No	350	89.7
**Ds good for health**		
Agree	299	76.7
Disagree	36	9.2
Don’t know	55	14.1
**Do you personally recommend use of dietary supplements to others**		
Yes, I always recommend	75	19.2
Yes, only when doctors recommend	219	56.2
Not at al	96	24.6

### Knowledge about dietary supplements among study participants

Assessment of knowledge about dietary supplements among Bangladeshi university students have shown in Table 2. The majority of the respondents believed (strongly agree/ agree) that food additives should be avoided (87.1%), and dietary supplements recommended by health professionals are effective (86.2%). Nearly half of the respondents (42.8%) dissented from the statement (either disagree or strongly disagree) that commercial dietary supplements were effective or accurate. Moreover, nearly three-quarters of the respondents either agreed or strongly agreed that they wanted to use dietary supplements that were made from food items (72.8%) and from natural ingredients or herbs that are safe (73.3%) (Table 2).

**Table 2:** Knowledge about dietary supplements among university students (N = 390).

Statements	Response, n (%)				
	Strongly Agree	Agree	Neither agree nor disagree	Disagree	Strongly Disagree
1. Dietary supplements are safe because they are just food items.	54 (13.8)	163 (41.8)	56 (14.4)	103 (26.4)	14 (3.6)
2. Dietary supplements made from natural ingredients or herbs are safe.	89 (22.8)	197 (50.5)	43 (11.0)	58 (14.9)	3 (0.8)
3. Food additives should be avoided.	194 (49.7)	146 (37.4)	30 (7.7)	12 (3.1)	8 (2.1)
4. Dietary supplements made from food items are safe.	85 (21.8)	199(51.0)	61 (15.6)	40 (10.3)	5 (1.3)
5. The efficacy of commercial dietary supplements is confirmed and reliable.	34 (8.7)	87 (22.3)	103 (26.4)	134 (34.4)	32 (8.2)
6. I want to use dietary supplements that have a good reputation.	73 (18.7)	193 (49.5)	59 (15.1)	51 (13.1)	14 (3.6)
7. Dietary supplements recommended by health professionals are effective.	124 (31.8)	212 (54.4)	31 (7.9)	20 (5.1)	3 (0.8)
8. Dietary supplements can prevent diseases.	62 (15.9)	157 (40.3)	106 (27.2)	57 (14.6)	8 (2.1)
9. Dietary supplements can compensate for an unbalanced diet.	60 (15.4)	165 (42.3)	88 (22.6)	71 (18.2)	5 (1.5)
10. Children who are picky eaters should take dietary supplements to supplement nutrition.	41 (10.5)	213 (54.6)	78 (20.0)	54 (13.8)	4 (1.0)
11. Pregnant women should take dietary supplements to supplement nutrition.	122 (31.3)	159 (40.8)	65 (16.7)	30 (7.7)	14 (3.6)
12. I want to use dietary supplements for weight loss or muscle building.	61 (15.6)	104 (26.7)	58 (14.9)	118 (30.3)	49 (12.6)

### Opinions and attitudes regarding dietary supplement (OADS)

As seen in Table 3, opinions and attitudes towards the use of dietary supplements were shaped by strongly agree being the most preferred answer. The majority of the respondents (80.5%) showed a strong consensus concerning use of dietary supplement only as per physician recommendation or harmful if not used properly. Above two-thirds of the respondents were positive about the importance of dietary supplement for health and wellbeing (69.2%), and in the context risk and adverse effect (67.9%).

**Table 3:** Opinions and attitudes regarding dietary supplement among study participants.

Opinion and attitudes of dietary supplement (OADS)	Response, n (%)				
	Strongly Disagree	Moderately Disagree	Neutral	Moderately Agree	Strongly Agree
1. It prevents chronic illness if used regularly.	46 (11.8)	52 (13.3)	93 (23.8)	158 (40.5)	41 (10.5)
2. Safe with minimal risk of adverse effects.	10 (2.6)	45 (11.5)	70 (17.9)	185 (47.4)	80 (20.5)
3. Prevents cancer.	72 (18.5)	76 (19.5)	117 (30.0)	89 (22.8)	36 (9.2)
4. Important for health and general well-being.	15 (3.8)	37 (9.5)	68 (17.4)	170 (43.6)	100 (25.6)
5. Use only as per physician recommendation/ harmful if not used properly.	18 (4.6)	26 (6.7)	32 (8.2)	137 (35.1)	177 (45.4)
6. Necessary for all ages.	68 (17.4)	91 (23.3)	83 (21.3)	86 (22.1)	62 (15.9)

### Prevalence and factors associated with the use of dietary supplement

The overall prevalence of dietary supplement use among the respondents was 15.6%. DS usage was reported as 3.33% in male, and 12.3% in females.

As shown in Table 4, the adjusted regression model revealed that female students were more likely to use DSs compared to male students (adjusted odds ratio, AOR = 5.44, 95% CI: 2.18–13.52). Respondents whose aged ≥25 years old were less likely to use DS compared to their counterparts (AOR = 0.08, 95% CI: 0.01–0.67). There was a greater likelihood of consuming DSs among those who suffered from a major illness (AOR = 6.99, 95% CI: 1.98–24.70) compared to non-sufferers. The likelihood of DS use was 5 times higher among those who had reported them as underweight compared to normal BMI (AOR = 5.855, 95% CI: 1.95–17.62). Respondents who agreed “DS is for good health” were more likely to take supplements compared to those who didn’t agree (AOR = 7.68, 95% CI: 1.66–35.58). Respondents who had a good level of knowledge about DS usage were more likely to use DS as compared to their counterparts (AOR = 2.64, 95% CI: 1.23, 5.64) (Table 4).

**Table 4:** Regression analysis showing the factors associated with the use of dietary supplement among Bangladeshi university students (N = 390).

Characteristics	Unadjusted model		Adjusted model		
	COR (95% CI)	p-value	AOR (95% CI)	p-value	VIF
**Age (years)**					1.606
18–20	Reference		Reference		
21–24	0.49 (0.26, 0.93)	**.030**	0.55 (0.22, 1.4)	.223	
25 and above	0.22 (0.06, 0.82)	**.024**	0.08 (0.01, .67)	**.020**	
**Gender**					1.174
Male	Reference		Reference		
Female	3.58 (1.87,6.86)	**< .001**	5.44 (2.18, 13.52)	**< .001**	
**Household income**					1.096
0–15000	Reference		Reference		
15001–30000	0.96 (0.38, 2.42)	.939	0.73 (0.23, 2.32)	.599	
30001–45000	1.45 (0.55, 3.82)	.445	1.54 (0.43, 5.48)	.507	
45001-above	2.66 (1.03, 6.88)	**.043**	1.68 (0.49, 5.76)	.412	
**Study year**					1.551
1^st^ year	Reference		Reference		
2^nd^ year	0.31 (0.13, 0.75)	**.009**	0.24 (0.07, 0.79)	**.019**	
3^rd^ year	0.56 (.26, 1.21)	.140	0.73 (0.25, 2.16)	.577	
4^th^ year	0.56 (0.25, 1.2)	.167	1.01 (0.31, 3.27)	.992	
above honors	0.49 (0.16, 1.5)	.217	4.67 (0.89, 24.47)	.068	
**Current marital status**					1.046
Married	0.58 (0.17, 1.90)	.381	0.29 (0.06, 1.39)	.122	
Unmarried	Reference		Reference		
**Suffer from a major Illness**					1.122
Do not suffer	Reference		Reference		
Suffer	4.57 (1.84, 11.39)	**.001**	6.99 (1.98, 24.70)	**.003**	
**Self-Related BMI**					1.091
Normal	Reference		Reference		
Overweight or obese	1.21 (0.61, 2.41)	.592	0.62 (0.25, 1.55)	.309	
Underweight	3.78 (1.75, 8.14)	**.001**	5.86 (1.95, 17.62)	**.002**	
**Self-rated health status**					1.606
Excellent	Reference		Reference		
Good	0.85 (0.34, 2.10)	.721	0.38 (0.12, 1.24)	.109	
Fair	1.63 (0.62, 4.32)	.323	1.05 (0.30, 3.69)	.934	
Below	2.91 (1.09, 7.73)	**.032**	2.58 (0.71, 9.33)	.148	
**Type of diet followed**					1.126
High Protein Diet	Reference		Reference		
Low fat diet	0.81 (0.19, 3.52)	.779	0.99 (0.15, 6.69)	.993	
No specific diet followed	1.52 (0.51, 4.49)	.451	1.86 (0.43, 8.12)	.408	
**Smoking status**					1.154
Current smoker	0.37 (0.09, 1.62)	.190	0.29 (0.04, 2.30)	.243	
Ex-smoker	0.78 (0.17, 3.55)	.748	1.72 (0.22, 13.40)	.605	
Never smoking	Reference		Reference		
**Physical Activity**					1.062
No activity	Reference		Reference		
<30 minutes/day	1.19 (0.64, 2.21)	.585	1.31 (0.61, 2.80)	.489	
>30 minutes per day	1.42 (0.68, 2.94)	.346	1.98 (0.76, 5.18)	.159	
**Diagnosis of chronic disease**					1.154
Yes	2.63 (1.25, 5.52)	**.010**	2.19 (0.75, 6.41)	.151	
No	Reference				
**Ds good for health**					1.177
Agree	3.91 (1.18, 12.97)	**.026**	7.68 (1.66, 35.58)	**.009**	
Disagree	1.58 (0.30, 8.28)	.591	1.86 (0.24, 14.16)	.549	
Don’t know	Reference		Reference		
**Knowledge of DS**					1.215
Bad	Reference		Reference		
Good	1.45 (0.83, 2.51)	.186	2.64 (1.23, 5.64)	**.012**	
**Attitude regarding DS**					1.209
Negative	Reference		Reference		
Positive	1.97 (1.13, 3.42)	**.017**	0.50 (0.241, 1.03)	.062	

Note: COR = crude odds ratio, AOR = Adjusted odds ratio, CI = Confidence interval and VIF = variance inflation factor. Bolded values indicates statistically significant (*p* < 0.05).

### Reasons and purpose for use of dietary supplement

Table 5 summarizes the prevalence, reasons, types, and sources of dietary supplements. Results revealed that 15.6% of respondents used DS during the last three months. In terms of DS users, the majority accessed DS for physician recommendations and for general health and well-being, followed by for weight gaining and for energy source. In some cases, students used supplements as a method of controlling hair, fall and skin care, as well as an energy booster for sports performance, or as a means of progressing athletically. In terms of consumption, iron and folic acid (IFA) tablets were the most popular, followed by calcium tablets and vitamin D among the all-other vitamins along with the multivitamin supplements. Families, friends, and acquaintances were the most likely sources of dietary supplement information, 3.58% and 3.33%, respectively, followed by newspapers, magazines, and flyers (2.56%), and finally in-store advertisements (1.79%).

**Table 5:** Reasons, types and sources for using dietary supplements among study participants.

Statements	Yes, n (%)	No, n (%)
**Do you use dietary supplement in the last three months?**	61 (15.6)	329 (84.4)
**Reason for usage of DS**		
Physician recommendations	30 (7.69)	31 (7.94)
General health and well being	30 (7.69)	31 (7.94)
For weight gaining	12 (3.07)	49 (12.5)
For growth	11 (2.82)	50 (12.82)
For weight loss	9 (2.30)	52 (13.33)
For energy source	16 (4.10)	45 (11.53)
Immune booster	18 (4.61)	43 (11.02)
Progress academic performance	8 (2.05)	53 (13.58)
Increase performance/sports or progress athletic performance	9 (2.30)	52 (13.33)
To control hair, fall and skin care	22 (5.64)	39 (10)
Increase endurance/body building	11 (2.82)	50 (12.82)
Memory enhancer	6 (1.53)	55 (14.10)
Other reasons (pregnancy-induced anemia and fatigue)	9 (2.30)	52 (13.33)
No reason mentioned	9 (2.30)	50 (12.82)
**Types of dietary supplement (TDS)**		
Multivitamins alone or in combination	18 (4.61)	43 (11.02)
Omega 3 fatty acid	6 (1.54)	55 (14.10)
Whey protein	12 (3.07)	49 (12.56)
Calcium	20 (5.12)	41 (10.51)
Vitamin-A	15 (3.84)	46 (11.79)
Vitamin-B	12 (3.07)	49 (12.56)
Vitamin-D	20 (5.12)	9 (2.30)
Vitamin-E	15 (3.84)	46 (11.79)
Vitamin-C	17 (4.35)	44 (11.28)
Iron and folic acid (IFA)	31 (7.94)	30 (7.69)
Other supplements (prescription and natural products)	18 (4.61)	43 (11.02)
**Sources**		
Internet	8 (2.05)	53 (13.58)
Social Media	8 (2.05)	53 (13.58)
Family	14 (3.58)	47 (12.05)
In-store advertisements	7 (1.79)	54 (13.84)
Product labels	8 (2.05)	53 (13.58)
Television	9 (2.30)	52 (13.33)
Pharmacists or drug store clerks	10 (2.56)	51 (13.07)
Friends or acquaintances	13 (3.33)	48 (12.30)
Clinic (physicians, pharmacists, dietitians)	44 (11.28)	17 (4.35)
Newspapers, magazines, flyers	10 (2.56)	47 (12.05)
Others	7 (1.79)	54 (13.84)

### Adverse reactions experienced from dietary supplements

Participants were asked whether DS had caused them any adverse reactions. A mere 1.3% of participants experienced adverse effects with DS. Among those who experienced unwanted effects, confusion, headaches, and hair loss were common adverse reactions.

## Discussion

This study sought to assess the prevalence of dietary supplement use and its associated risk factors among university students in Bangladesh. The overall prevalence of dietary supplement use among the respondents was 15.6%. This figure was, however, below the prevalence of 41% reported from a study conducted among adult population in Southern Bangladesh [7]. An earlier study of pharmacy students in Pakistan found that 38.3% of them used DS [18]. In accordance with another study conducted in Bangladesh, female students at private universities were found to use DS more frequently [9]. The sociodemographic differences in study sites, sample sizes, and students’ academic backgrounds—whether they are in health-related fields or not—could be the reason of the discrepancies in the prevalence of DS usage. Generally, public universities have low educational expenses and majority of the students come from low-and middle-income families; therefore, DS usage among students of public universities may be lower than in other contexts or subjects.

The majority of the respondents (80.5%) showed a strong consensus concerning the use of dietary supplements only as per physician recommendations or harmful if not used properly. Meanwhile, in a previous study in Southern Bangladesh, the majority of participants consumed DS without physician recommendation [7]. This finding provides an opportunity for further probing in future to estimate if there is a gap between knowledge of DS and actual practice. Above two-thirds of the respondents were positive about the importance of dietary supplement for health and wellbeing (69.2%), and in the context of risk and adverse effects (67.9%). This finding was again in contrast to findings from a cross-sectional study in Dhaka City, Bangladesh, where more than half of the study participants had no knowledge about the value of DS [5]. The disparity in findings could be as a result of the level of education of the participants in each of the study areas. For instance, while the study participants in this study were university students, the study participants in the previous study were female hospital patients in Dhaka City.

In the present study, a prevalence of DS use was 3.33% in male students, and 12.3% in females. In line with this finding, the adjusted regression model revealed that female students were more likely to use DSs compared to male students. This supports the findings from previous studies [5, 7, 19–21]. This finding can be justified by the fact that females are more susceptible to suffering from nutritional deficiencies than males due to their biological reproductive system, low socioeconomic status, socio-cultural traditions and disparities in household work patterns and expectations which further predisposes females to malnutrition [5, 7].

Respondents aged ≥25 years were less likely to use DS compared to their counterparts. Age has been reported widely as one of the factors that influence DS use. It was documented before that young age is characterized by growth spurt or rapid physical growth supported by a balanced intake of nutrients [11]. However, due to different life styles of young people among other factors, many chose to take DS to balance their nutritional needs. It is therefore not surprising that in this study, respondents aged less than 25 years were more likely to use DS. In another study involving young college students, they used DS mainly for physical performance [22] and as such it is possible that undergraduate students in our study who may be in the same age bracket could be motivated by the same. This finding is however, in contrast with findings from a study in Southern Bangladesh where the likelihood of using DS increased with age [7].

There was a greater likelihood of consuming DS among those suffered from a major illness compared to non-sufferers. Persons with major illnesses usually require nutritional support and this often serves as the motivation for DS use. Others are advised by their physicians to use based on their nutritional deficits and requirements. This finding is in agreement with findings from a study in Saudi Arabia which reported that major illness was one of the motivations for DS use among students [1]. In another study in Northern Iran, participants with a history of obesity, hypertension, cancer and other major illness had greater odds of using DS [23]. Islam et al. (2018) [7] has also reported that many people in Bangladesh use DS for the treatment of illnesses.

The likelihood of DS use was 5 times higher among those who had reported them as underweight compared to students who had normal BMI. About 3% of our study participants reported that they use DS for weight gain and that plausibly explains this finding. Meanwhile, a previous study by Islam et al. (2018) [7] found that adults in Bangladesh who were underweight were less likely to use DS. This is in contrast with our study finding and the plausible reason could be the type of respondents recruited in each study. Whereas participants of our study were university students from selected universities in Southern Bangladesh, the participants of the previous study by Islam et al. (2018) [7] were adults in Southern Bangladesh irrespective of their education, occupation, and etc. but excluding pregnant women.

Families, friends, and acquaintances were the most common sources from where participants obtained DS-related information, followed by newspapers, magazines, and flyers, and finally in-store advertisements. This finding is quite concerning because the likely sources of DS information found in this study may not be from well-trained experts who may well inform and educate the students on the DS with scientific facts and evidence for informed decision making. There could be challenges with proper diagnosis by these sources which may lead to abuse of such DS.

Participants were asked if they had experienced any negative effects from using DS. Only 1.3% of the subjects experienced adverse effects with DS. Confusion, headaches, and hair loss were common adverse reactions among those who had side effects. This finding is consistent with report from a study among college students in Lagos Nigeria, where only 10% of DS users reported adverse reactions such as headaches, nausea, vomiting and dizziness [11]. Adverse reactions from DS are rare, but it is important for students to seek DS recommendations from physicians before use so that adverse reactions can be well managed.

The majority (7.7%) stated the reason for using DS as physician recommendations and for general health and well-being, followed by for weight gain (3.07%) and for energy sources (4.10%). In some cases, students used supplements as a method of controlling hair, fall and skin care (5.64%), as an energy booster for sports performance, or as a means of progressing athletically (2.30%). Reasons given by participants for DS use are similar to reasons expressed by students in Lagos Nigeria [11], undergraduate female students in Bangladesh [9] and adult population in southern Bangladesh [7]. In terms of consumption, IFA tablets were the most popular (7.9%), followed by calcium tablets (5.12%) and vitamin D (5.12%) among the all-other vitamins along with the multivitamin supplements (3.9%). Vitamins are widely used as DS because of their ability to boost one’s immunity and treat many diseases [22]. It is important to suggest that students need to be encouraged to seek recommendations from physicians before using DS.

### Limitations and strength of the study

The study employed a cross-sectional study design; therefore causality cannot be claimed, although we sought to ascertain the reasons why some of the students use DS. There is also a possibility of social desirability bias since the variables were self-reported. Since only two public institutions from the Barishal division were included in the study, the findings cannot be generalized to other public universities, all private universities, or the rest of the country. To obtain concrete and comprehensive results, a nationwide institution-based survey of DS use among university students at both public and private universities is highly recommended. Despite these limitations, the study has some important public health implications and also recommends some major aspects regarding DS usage. To the best of our knowledge, this is the first study in Bangladesh which investigates dietary supplement usage among both male and female students of public universities in Bangladesh. Moreover, the study’s findings may be used by the policymakers to improve DS usage among students. The study also employed validated tools making the findings more valid and reliable.

## Conclusion

The study revealed that a significant proportion of students are using DS where only 7.7% of them used DS according to physicians’ recommendations. Additionally, students used DS for general health and well-being, weight gaining and as a source of energy for physical and sporting activities, etc. Participants showed a strong consensus concerning the usage of DS according to physician recommendation or the harmfulness of DS usage if not used properly. Female students, those who were under weight, those with major illness and those with good knowledge about DS had more likelihood of using DS. The study revealed that students gathered knowledge about DS usage from different sources other than professional personnel. Thus, it’s recommended that an appropriate program should be adopted by the Ministry of Health and Family Welfare together with the Ministry of Education and the University Authorities in Bangladesh institute to educate students on proper and safer ways of using DS. Furthermore, there should be advocacy on university campuses so that students can obtain DS from only qualified and well-trained health professionals instead of family, friends and other acquaintances.

## Supporting information

S1 Data (ZIP)
